# A Suspected Case of Lyme Borreliosis in a Dog from Belgium

**DOI:** 10.1155/2019/3973901

**Published:** 2019-03-28

**Authors:** Marina Gatellet, Sébastien Vanderheyden, Michel Abee, Łukasz Adaszek, Marie Varloud

**Affiliations:** ^1^Ceva Santé Animale, Libourne, France; ^2^Cabinet vétérinaire Vanderheyden, Pepinster, Belgium; ^3^Ceva Santé Animale Benelux, Brussels, Belgium; ^4^University of Life Sciences, Lublin, Poland

## Abstract

A 5-year-old Jack Russell Spaniel was presented in December 2017 to his veterinarian in Belgium for sudden weakness, reluctance to move, and pain. Blood analysis showed no deviations and serum increased levels of* B. burgdorferi* s.l. antibodies were detected. The dog recovered a few days after the onset of doxycycline treatment. This case illustrates the possible relationship between tick-borne diseases and orthopedic problems.

## 1. Introduction

Borreliosis is a tick-borne disease caused by a gram-negative spirochete,* Borrelia burgdorferi *sensu lato* (B. burgdorferi *s.l.) which includes at least 11 subspecies, of which 3 are considered pathogenic for dogs:* Borrelia burgdorferi* sensu stricto (*B. burgdorferi *s.s.),* Borrelia afzelii*, and* Borrelia garinii*. Pathogenicity of other species has not been demonstrated so far. The vectors of* Borrelia* in Europe are* Ixodes* ticks which primarily live in deciduous woodland and mixed forest. These ticks can feed on various species of mammals and birds. In some European areas, up to 75% of* Ixodes* ticks can be infected by* Borrelia* spp. [1, 2]. This result might explain why, in Belgium, 21.6% of forest workers were detected seropositive for* Borrelia burgdorferi* [3]. Based on this observation, it is likely that dogs that often walk in the forest are also heavily exposed to spirochetes. In experimental studies, around 57% of* Borrelia* infected dogs exhibited clinical signs of Lyme disease [4–9]. Numbers appear to be much lower under field conditions, possibly because transitory clinical signs might be overlooked by pet owners. Clinical signs of Lyme borreliosis in dogs living in Europe were already described in the United Kingdom [10], Czech Republic [11, 12], Spain [13], Italy [14], Belgium [15], and Poland [16]. We report here a suspected case of Lyme borreliosis in a Belgian dog used to do long hikes in the forest. The dog was identified using social media where its owner reported the disease on the dog's personal webpage. A consent form was signed by the owner before investigating the case.

## 2. Case Description

The presented patient is a 5-year-old male Jack Russell Terrier and it is considered as a member of the family, lives mainly indoors, and is correctly vaccinated against distemper, parvovirosis, leptospirosis, Rubarth disease, parainfluenza, and rabies. The dog is treated monthly against external parasites with afoxolaner. The dog and owner live in Belgium (province of Liège). They are used to do long recreational hikes in the forest all year long and ticks were occasionally found and removed from its skin. The dog has no history of travelling abroad.

In December 2017, the owner observed sudden weakness and a reluctance of his dog to move from his basket. The following day, because of the persistency of the signs, the dog was presented to the local veterinarian who noticed a reluctance to move, generalized muscular pain, hindquarters articular pain, and a mild fever. Results of hematology examination and biochemistry were unremarkable. Serum was sent to an external laboratory only for serological examinations for* Toxoplasma gondii* and* Borrelia* spp.: the results of serology for toxoplasmosis were negative and Lyme serology (immunofluorescent antibody testing) was positive (1: 1024). As soon as the results were available, the dog was prescribed doxycycline 5 mg/kg* per os* twice a day for 30 days. Three days after initiation of the treatment, the dog's condition improved, the clinical signs disappeared and, from that moment, it never presented similar symptoms again.

## 3. Discussion

The presented clinical case illustrates systemic and orthopedic disorders as the main clinical manifestation of Lyme disease. Indeed, 53% of European cases mentioned musculoskeletal disorders. In this particular case, the owner did not observe lameness. This is, however, most likely due to the difficulty to observe lameness when both front and hind limbs are equally affected. In addition to orthopedic problems, pyrexia is detected in about 50% of the cases. Recovery is usually achieved within one month of antibiotic treatment, illustrating good prognosis of this clinical form.

Cases of canine borreliosis were already reported in two dogs in Belgium [15]. Similarly to our present case, both dogs exhibited orthopedic signs with lameness and loss of appetite. Suspicion was strengthened with ELISA and immunofluorescent antibody testing results. One dog developed paralysis of nerves V, VII, IV, and X which is a very rare condition.

The prevalence of* Borrelia* infection in ticks is a major component of risk-based assessment in human and veterinary medicine. A Belgian study shows that 12.0% of ticks are infected by* B. burgdorferi* s.l. with the majority being* B. afzelii* (55%) and* B. garinii* (21%) [17], and 8% were coinfected by at least two* Borrelia* species. Ruyts et al. [18] showed that 17.6% of nymphs were infected, most commonly by* B. afzelii*. Borreliosis should therefore be suspected in all cases of orthopedic problems and systemic disorders, particularly if forest hikes and tick infestation are reported by the owner. Moreover, limb dysfunction and systemic signs are the only symptoms validated by Koch's postulates, establishing a causal link between a pathogen and the disease. Other clinical manifestations, especially renal and cardiac, were previously described, but not yet reproduced experimentally, therefore remaining unvalidated as a clinical symptom of Lyme disease.

In case of orthopedic signs, such as lameness (including intermittent or shifting), pain, and joint swelling, other major causes should be excluded and the following tests should be considered: tibial-femoral movement to evaluate the anterior cruciate ligament (drawer sign) and imaging of swollen joints. If there are no abnormalities, blood analysis should be performed to evaluate systemic diseases, if possible combined with measuring creatine kinase to detect polymyositis. To evaluate arthritis, arthrocentesis is indicated with cytology of the synovial fluid and PCR to detect* Borrelia* DNA. Other infections are to be considered, such as anaplasmosis, ehrlichiosis, or immune-mediated polyarthritis. In the case presented, no radiographs were performed because of the absence of a trauma history; in combination with the detected fever, a systemic process was suspected. As lameness is not a pathognomonic sign, serology and, if possible, evidence of pathogen presence with polymerase chain reaction (PCR) is indicated.

In this case, serology demonstrated exposure to the pathogen, but this is not predictive of developing clinical signs and the antibody level is not correlated to the severity of the symptoms [4]. There are a lot of serology methods available today. ELISA, immunofluorescent antibody (IFA), or Western Blot is not recommended as a single test because of the possibility of cross-reactions with other spirochetal exposure [19]. Some tests, e.g., quantitative C6 ELISA (Idexx), are based on the detection of antibodies to the C6 peptide, highly specific for* B. burgdorferi* s.l. and expressed only during a natural infection. In addition to being more specific, this test also allows differentiation between vaccinated and nonvaccinated dogs [20]. Moreover, this test was demonstrated to be highly sensitive and specific (100%) for possible early detection of the pathogen (30% of dogs three weeks after initial infection) [20]. Levy et al. [21] demonstrated the median drop off of this quantitative value 6 months after treatment was 68%, depending on the initial level. This test is thus useful for the follow-up. PCR in blood often provides false negative results as bacteremia in case that borreliosis seems not permanent [22]. PCR sensitivity of synovial fluid seems to be quite high according to some veterinarians, but it is not supported experimentally.

Doxycycline is the drug of choice in the therapy of canine Lyme disease, but at its normal dose (10 mg/kg once a day for a least one month) it may trigger gastrointestinal side effects [23]. Dividing the dose in two administrations of 5 mg/kg per day can limit these adverse effects. As doxycycline is not always well tolerated or in case of young animals, other possibly effective antibiotics are indicated: minocycline 12 mg/kg twice a day or 25 mg/kg once a day during at least 1 month, amoxicillin 20 mg/kg three times a day for at least one month, azithromycin 25 mg/kg once a day for 1 month minimum, or chloramphenicol in case of neurologic disorders 15-25 mg/kg three times a day for 14-30 days.

In most dogs with orthopedic borreliosis, the prognosis is good. But some treated animals remain nonclinical carriers [19]. Some of these dogs can exhibit similar clinical signs months to years later because of a new infection (dogs are not protected after an infection) or reactivation of the pathogen.

As a conclusion, in case of orthopedic problems when no traumatic cause can be identified, Lyme disease should be considered, especially in endemic area or when travel to an endemic area was reported. To support any suspicion and to facilitate follow-up, serology (especially quantitative C6-ELISA testing) is strongly recommended. If possible (and indicated), a synovial fluid PCR should be considered. After antibiotic administration and, if needed, analgesic treatment, most dogs will recover. However, pet owners should be aware that their dog is not protected and reoccurrence can happen.

## Supplementary Materials

Supplementary Materials The video shows the dog at the onset of his clinical signs.
